# Radiomic Features of the Nigrosome-1 Region of the Substantia Nigra: Using Quantitative Susceptibility Mapping to Assist the Diagnosis of Idiopathic Parkinson's Disease

**DOI:** 10.3389/fnagi.2019.00167

**Published:** 2019-07-16

**Authors:** Zenghui Cheng, Jiping Zhang, Naying He, Yan Li, Yaofeng Wen, Hongmin Xu, Rongbiao Tang, Zhijia Jin, E. Mark Haacke, Fuhua Yan, Dahong Qian

**Affiliations:** ^1^Department of Radiology, Ruijin Hospital, Shanghai Jiao Tong University School of Medicine, Shanghai, China; ^2^School of Biomedical Engineering, Shanghai Jiao Tong University, Shanghai, China; ^3^Department of Radiology, Wayne State University, Detroit, MI, United States; ^4^Institute of Medical Robotics, Shanghai Jiao Tong University, Shanghai, China

**Keywords:** nigrosome-1, substantia nigra, Parkinson's disease, quantitative susceptibility mapping, radiomics, support vector machine

## Abstract

**Introduction:** The loss of nigrosome-1, which is also referred to as the swallow tail sign (STS) in T2^*^-weighted iron-sensitive magnetic resonance imaging (MRI), has recently emerged as a new biomarker for idiopathic Parkinson's disease (IPD). However, consistent recognition of the STS is difficult due to individual variations and different imaging parameters. Radiomics might have the potential to overcome these shortcomings. Therefore, we chose to explore whether radiomic features of nigrosome-1 of substantia nigra (SN) based on quantitative susceptibility mapping (QSM) could help to differentiate IPD patients from healthy controls (HCs).

**Methods:** Three-dimensional multi-echo gradient-recalled echo images (0.86 × 0.86 × 1.00 mm^3^) were obtained at 3.0-T MRI for QSM in 87 IPD patients and 77 HCs. Regions of interest (ROIs) of the SN below the red nucleus were manually drawn on both sides, and subsequently, volumes of interest (VOIs) were segmented (these ROIs included four 1-mm slices). Then, 105 radiomic features (including 18 first-order features, 13 shape features, and 74 texture features) of bilateral VOIs in the two groups were extracted. Forty features were selected according to the ensemble feature selection method, which combined analysis of variance, random forest, and recursive feature elimination. The selected features were further utilized to distinguish IPD patients from HC using the SVM classifier with 10 rounds of 3-fold cross-validation. Finally, the representative features were analyzed using an unpaired *t*-test with Bonferroni correction and correlated with the UPDRS-III scores.

**Results:** The classification results from SVM were as follows: area under curve (AUC): 0.96 ± 0.02; accuracy: 0.88 ± 0.03; sensitivity: 0.89 ± 0.06; and specificity: 0.87 ± 0.07. Five representative features were selected to show their detailed difference between IPD patients and HCs: 10th percentile and median in IPD patients were higher than those in HCs (all *p* < 0.00125), while Gray Level Run Length Matrix (GLRLM)-Long Run Low Gray Level Emphasis, Gray Level Size Zone Matrix (GLSZM)–Gray Level Non-Uniformity, and volume (all *p* < 0.00125) in IPD patients were lower than those in HCs. The 10th percentile was positively correlated with UPDRS-III score (*r* = 0.35, *p* = 0.001).

**Conclusion:** Radiomic features of the nigrosome-1 region of SN based on QSM could be useful in the diagnosis of IPD and could serve as a surrogate marker for the STS.

## Introduction

Parkinson's disease (PD) is a common neurodegenerative movement disorder, and mainly characterized by a loss of dopaminergic neurons and iron accumulation in the substantia nigra (SN), pathologically (Damier et al., 1999). The dopaminergic neuronal loss and iron deposition were reported to occur in nigrosomes of the SN pars compacta (SNpc) at the initial stage of PD, especially in the nigrosome-1, the largest among the five subdivisions (Damier et al., 1999; Lehericy et al., 2014). Therefore, imaging the nigrosome-1 using iron-sensitive magnetic resonance imaging (MRI), for example, T2^*^-weighted imaging, has recently been investigated and validated as a possible biomarker for idiopathic Parkinson's disease (IPD) (Noh et al., 2015; Reiter et al., 2015).

Recognizing the nigrosome-1 has been possible thanks to the presence of high iron signal surrounding it that produces what is referred to as the “swallow tail sign” (STS) (Schwarz et al., 2014). The loss of the STS is thought to be due to the increase in iron content subsequent to the depigmentation of the neuromelanin in the nigrosome-1 territory. The reported sensitivity, specificity, and accuracy of the loss of nigrosome-1 hyperintensity or the STS varied from 94 to 100%, 84.6 to 94.4%, and 94.6 to 96%, respectively (Noh et al., 2015; Reiter et al., 2015; Mahlknecht et al., 2017; Stezin et al., 2018). However, consistent recognition of the STS among reviewers has been difficult due to individual differences in the shape of the nigrosome-1 territory and to the choice of imaging parameters such as scanning plane, resolution, signal-to-noise ratio (SNR), and echo time, even on the ultra-high 7.0 T system MRI (Schmidt et al., 2017; Kim et al., 2018).

Conventional quantitative imaging (e.g., R2^*^) of SN was reported to have the potential to differentiate PD from healthy controls (HCs) by quantification of the local iron content (Martin et al., 2008; Du et al., 2011), which partially avoids some of the shortcomings referred above. Nevertheless, the quantification can be affected by several factors including intravoxel spin dephasing, non-locality of phase and tissue susceptibility, as well as field strength (Schweser et al., 2011; Li et al., 2012; He et al., 2015). Quantitative susceptibility mapping (QSM) has been proven to be a sensitive and reliable quantitative method reflecting the local tissue susceptibility with much better contrast compared to R2^*^ or T2 methods (Langkammer et al., 2012; He et al., 2015; Murakami et al., 2015; Wang and Liu, 2015; Du et al., 2016). However, diagnostic accuracy did not surpass that of conventional imaging, which might be accredited to the intergroup overlap of the susceptibility value when utilizing the mean value of a given structure (Kim et al., 2018).

Radiomics is a recently developed promising technique, which can be utilized to mine feature information (such as density, shape, size, and texture) of a certain region of interest underlying medical images and it can generate a great deal of quantitative features including some reflecting the inter-voxel spatiality (Feng et al., 2018). Radiomics has been mainly applied to various tumors, and its role in diagnosis, treatment evaluation, and prognosis has to some degree been verified (Cameron et al., 2016; Nie et al., 2016; Zhang et al., 2017).

To date, few investigations of radiomics on neurodegenerative disease have been carried out. We hypothesized that it might have the potential to overcome and/or complement the shortcomings of the current nigrosome-1 imaging referred to above. Therefore, our purpose was to explore whether radiomic features of the iron content in the nigrosome-1-containing part of the SN based on QSM data could help to differentiate IPD patients from HCs.

## Materials and Methods

### Participants

This study was approved by our institutional review board and written informed consent was given and signed by all the participants. The diagnosis of IPD was based on the UK Parkinson's Disease Society Brain Bank Clinical Diagnostic Criteria (Hughes et al., 1992). The exclusion criteria were as follows: (1) secondary or atypical parkinsonism; (2) dementia: Mini-Mental State Examination (MMSE) score <24; (3) a history of cerebrovascular disease (e.g., infarction, hemorrhage), brain tumor, head trauma, or any other type of psychiatric disorders; (4) a history of medication known to cause parkinsonism or affect clinical assessment; or (5) contraindications to an MRI examination. HCs with gender and age matched from local communities were recruited according to the following inclusion criteria: (1) older than 40 years, without family history of movement disorders; (2) without any neurological or psychiatric disorders; and (3) an MMSE score of at least 24. There were 87 IPD patients (41 males and 46 females, aged 60.9 ± 8.1 years) and 79 HCs (43 males and 34 females, aged 63.4 ± 7.3 years) enrolled according to the above criteria from March 2012 to June 2015. Two HCs with imaging quality score higher than two were excluded for further analysis (see the grading system in the section Image Reconstruction).

### MRI Acquisition

All participants were scanned on a 3.0-T MRI system (Signa HDxt; GE Healthcare, Milwaukee, WI, USA) equipped with an eight-channel phased-array head coil. Foam pads and earplugs were applied to reduce head movement and scanner noise, respectively. A three-dimensional multi-echo gradient-echo (GRE) sequence was used to generate T2^*^-weighted images with the following parameters: TR = 59.3 ms; TE1 = 2.7 ms, ΔTE = 2.9 ms, number of echoes = 16, flip angle = 12°, FOV = 22 × 22 cm^2^, matrix = 256 × 256, resolution = 0.86 × 0.86 × 1.0 mm^3^, bandwidth = 488.28 Hz/pixel, acceleration factor = 2, number of slices = 136, and acquisition time = 10 min 42 s. In addition, conventional sequences including T1-weighted images, T2-weighted fluid-attenuated inversion recovery (FLAIR) images, and diffusion-weighted imaging (DWI) were also acquired to screen for cerebrovascular diseases and other confounding diseases. The whole brain was covered axially parallel to the anterior commissure–posterior commissure (AC–PC) line.

### Image Reconstruction

Before reconstruction of QSM, two neuroradiologists (ZC, NH) blindly assessed the quality of magnitude images using the following grading system (He et al., 2015): 1 = very good (little or no artifact); 2 = good (visible artifacts); 3 = poor (considerable motion artifacts); 4 = very poor (significant motion artifacts), 5 = non-diagnostic scan, and reached a consensus by discussion on the disagreements. Subjects with a score higher than two were excluded from further processing. Of the 79 HCs, 75 cases were scored 1; 2 and 2 cases were scored 2 and 3, respectively. Of the IPD cases, 85 cases scored 1 and the remaining 2 cases scored 2. QSM reconstructions were performed utilizing the method reported previously (Li et al., 2011). A brief description follows: (1) phase images were collected from each channel of the coil, and then averaged; (2) background phase variations were removed using SHARP (Sophisticated Harmonic Artifact Reduction for Phase) with a filter radius of 8 (Schweser et al., 2011); and (3) the susceptibility map was generated using an improved LSQR (iLSQR) method (Li et al., 2011, 2015) with the regularization threshold for Laplace filtering being set at 0.04.

### Image Analysis and Segmentation

**Figure 1:**
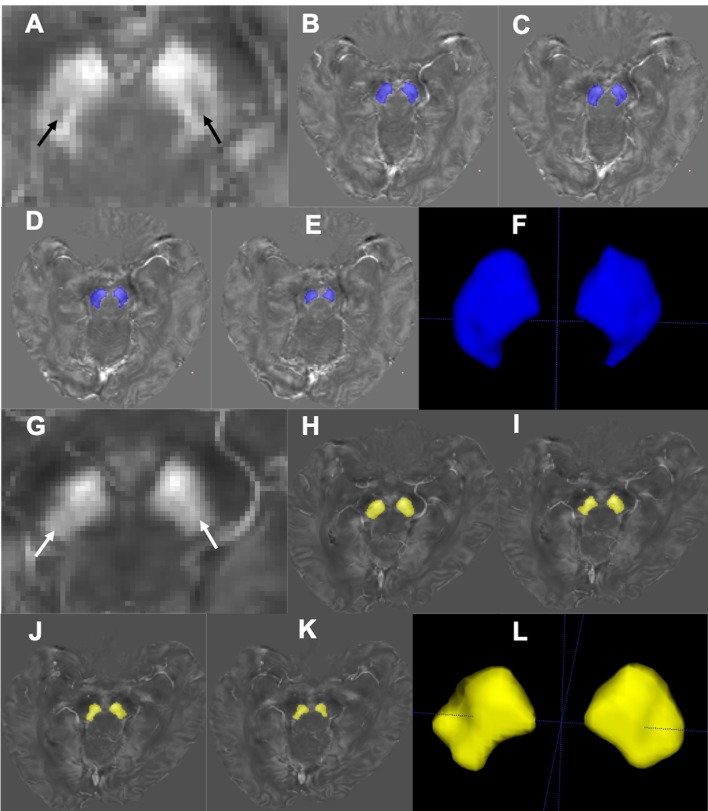
Segmentation of the nigrosome-1-containing region of SN. Representative QSM images of the nigrosome-1 area for a HC (67 years old, male; **A**) and an IPD patient (69 years old, male, H&Y = 2; **G**). The nigrosome-1 area presents as the “swallow tail” sign bilaterally (**A**, black arrow) in the HC, while it cannot be seen in the IPD case (**G**, white arrow) (that is, there is a loss of the “swallow tail” sign). Regions of the SN below the RN were segmented slice by slice **(B–E**; **H–K)** to generate the three-dimensional nigrosome-1-containing SN **(F,L)**.

The STS is composed of the nigrosome-1 (hyperintensity on SWI) and its surrounding structures (hypointensity on SWI) (Schwarz et al., 2014). This usually appears at or below the caudal part of the red nucleus (RN) (Massey et al., 2017). This nigrosome-1-containing territory was set as the region of interest (ROI) and the relevant slices were manually drawn and segmented on the QSM data using ITK-SNAP (V3.4.0, http://www.itksnap.org) by one of the investigators (J.Z) blindly from the level of the inferior part of the RN to the inferior part of the SN (Figure 1). The most inferior part and the boundary regions of the SN were excluded to avoid partial volume effects. The lateral and medial parts of SN were delineated on a visible border of high-intensity signal of SN on QSM, with boundary voxels excluded. The final volume of interest (VOI) consisted of four slices unilaterally, and the bilateral VOIs were confirmed by a neuroradiologist (Z.C., with 5 years' experience on neuroimaging diagnosis).

As a comparison, all the subjects were reviewed blindly on QSM for manifestation of STS or not by a neuroradiologist (ZC, with 5 years' experience on neuroimaging diagnosis). Bilateral and unilateral loss of the sign were classified as positive, and bilateral presence of the sign was classified as negative.

### Feature Extraction

One hundred and five radiomic features including 18 first-order features, 13 shape-based features, and 74 texture features were extracted from the segmented nigrosome-1-containing SN images. The first-order features describe how the individual susceptibilities are distributed, consisting of mean, 10th percentile, variance, and 15 other features. The shape-based features describe the geometric characteristics of the VOI, such as volume, surface area, and diameter. The textural features express how individual susceptibilities distribute spatially, which are computed according to five defined matrices: Gray Level Co-occurrence Matrix (GLCM), Gray Level Dependence Matrix (GLDM), Gray Level Run Length Matrix (GLRLM), Gray Level Size Zone Matrix (GLSZM), and Neighboring Gray Tone Difference Matrix (NGTDM). The (*i, j*)th element of GLCM represents the frequency of the combination of gray levels *i* and *j* of two pixels, which are separated by a distance of δ along angle θ; GLDM quantifies gray-level dependencies in an image, and gray-level dependency is defined as the number of connected voxels that are dependent on the center voxel within distance δ along angle θ; GLRLM quantifies gray-level runs, which are defined as the length of consecutive pixels that have the same gray-level value within distance δ along angle θ; GLSZM quantifies gray-level zones in an image, and a gray-level zone is defined as the number of connected voxels that share the same gray-level intensity. The NGTDM quantifies the difference between a gray value and the average gray value of its neighbors within distance δ. In our study, we considered one neighboring pixel (δ = 1). As for the parameter angle θ in GLCM, GLDM, GLRLM, we consider all 13 possible directions as (1, 0, 0), (0, 1, 0), (0, 0, 1), (1, 1, 0), (1, 0, 1), (0, 1, 1), (1, −1, 0), (−1, 1, 0), (1, 0, −1), (−1, 0, 1), (0, −1, 1), (0, 1, −1), and (1, 1, 1) for our volumetric data, and the mean values of the features form different directions were used as the final features.

The meaning and equation of each feature from the above mentioned seven groups (first-order features, shape-based features, and textural feature-based GLCM, GLDM, GLRLM, GLSZM, and NGTDM) were described thoroughly in Supplementary S1, and 17 representative features were listed in Table 1. The feature extraction procedure was performed using the open-source python package pyradiomics (Jjm et al., 2017). All the feature descriptions are consistent to the illustration in their website (https://pyradiomics.readthedocs.io/en/latest/index.html).

**Table 1:** Description and equation of the 13 representative features from different group.

**Features**	**Description**	**Equation**
Firstorder-minimum	The minimum susceptibility in the VOI	min(X)
Firstorder-10 Percentile	The 10th percentile of the sorted susceptibility in the VOI	10th_percentile(X)
Median	Median of the sorted susceptibility in the VOI	Median(X)
GLCM-Correlation	The image complexity	$\frac{\overset{N_{g}}{\underset{i=1}{\sum }}\overset{N_{g}}{\underset{j=1}{\sum }}p\left(i,j\right)ij-\mu_{x}\mu_{y}}{\sigma_{x}\left(i\right)\sigma_{y}\left(j\right)}$
GLCM-Informational Measure of Correlation 1 (InfMCor1)	Complexity of the texture	$\frac{HXY-HXY1}{max\left\{HX,HY\right\}}$
GLCM-SumEntropy (SumEntrp)	The sum of neighborhood intensity value differences	$\overset{2N_{g}}{\underset{k=2}{\sum }}p_{x+y}\left(k\right){log}_{2}\left(p_{x+y}\left(k\right)\right)$
GLDM-Dependence Entropy (DepdEntrp)	The randomness of GLDM. Higher Dependence Entropy implies more complex texture	_p(**i*, *j**)*log*_2_(**p**(**i*, *j**))_
GLDM-Dependence Variance (DepdVar)	The variance in dependence size. Higher Dependence Variance implies more heterogeneity in local zone size.	$\overset{N_{d}}{\underset{j=1}{\sum }}p\left(i,j\right){\left(j-\mu \right)}^{2}\text{,where}\;\mu =\overset{N_{g}}{\underset{i=1}{\sum }}\overset{N_{d}}{\underset{j=1}{\sum }}jp\left(i,j\right)$
GLDM-GrayLevel Non-Uniformity (GryLvNon-Uni)	The similarity of gray-level intensity values in the image. A lower GLN value correlates with a greater similarity in intensity values	$\frac{\overset{N_{g}}{\underset{i=1}{\sum }}{\left(\overset{N_{d}}{\underset{j=1}{\sum }}\text{P}\left(i,j\right)\right)}^{2}}{N_{z}}$
GLDM-Dependence Non-uniformity Normalized (DepdNonUni)	The similarity of dependence throughout the image, with a lower value indicating more homogeneity among dependencies of the image.	$\frac{\overset{N_{d}}{\underset{j=1}{\sum }}{\left(\overset{N_{g}}{\underset{i=1}{\sum }}\text{P}\left(i,j\right)\right)}^{2}}{N_{z}^{2}}$
GLRLM-RunEntropy (RunEntrp)	The randomness of run lengths and gray levels. A higher value indicates more heterogeneity in the texture patterns	_p(**i*, *j**)*log*_2_(**p**(**i*, *j**))_
GLRLM-RunLengthNonUniformityNormalized (RunLthNonUni)	The similarity of run lengths throughout the image. A lower value indicates more homogeneity among run lengths of the image	$\frac{\overset{N_{r}}{\underset{j=1}{\sum }}{\left(\overset{N_{g}}{\underset{i=1}{\sum }}\text{P}\left(i,j\right)\right)}^{2}}{{N_{r}}^{2}}$
GLRLM-HighGrayLevelRunEmphasis (HGLRunEmphs)	The distribution of the higher gray-level values, with a higher value indicating a greater concentration of high gray-level values in the image	$\frac{\overset{N_{g}}{\underset{i=1}{\sum }}\overset{N_{r}}{\underset{j=1}{\sum }}\text{P}\left(i,j\right)i^{2}}{N_{r}}$
GLRLM-LongRunLowGrayLevelEmphasis (LRunLGREmphs)	The joint distribution of long run lengths with lower gray-level values	$\frac{\overset{N_{g}}{\underset{i=1}{\sum }}\overset{N_{r}}{\underset{j=1}{\sum }}\frac{\text{P}\left(i,j\right)j^{2}}{i^{2}}}{N_{r}}$
GLSZM-ZoneEntropy (ZoneEntrp)	The randomness in the distribution of zone sizes and gray levels. A higher value indicates more heterogeneity in the texture patterns	*p*(*i, j*)log_2_(*p*(*i, j*))
GLSZM-GrayLevelNonUniformity (GryLvNonUniS)	The variability of gray-level intensity values in the image, with a lower value indicating more homogeneity in intensity values	$\frac{\overset{N_{g}}{\underset{i=1}{\sum }}{\left(\overset{N_{s}}{\underset{j=1}{\sum }}\text{P}\left(i,j\right)\right)}^{2}}{N_{z}}$
Shape-Volume	The volume of the VOI	The voxel number in VOI

*The first part of the feature name is its group. The X is the susceptibility in the VOI; N_g_ is the number of discrete intensity values in the VOI; P(i, j) is the value in corresponding matrix; N_d_ and N_z_ are the number of discrete dependency sizes and the number of dependency zones in GLDM; N_r_ is the number of discrete run lengths in the GLRLM; μ, σ, HX, HY, and HXY denote the mean, standard deviations, entropy of p_x_, entropy of p_y_, and entropy of p(i,j) in GLCM, respectively. More details can be found in the Supplementary S1 or the abovementioned pyradiomics website*.

### Feature Selection

**Figure 2:**
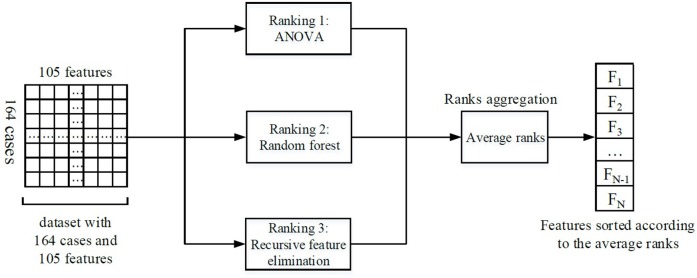
Illustration of the feature selection process. Firstly, three feature selection methods ranked the features individually. Then, the ranks from different methods were averaged. Finally, the features were sorted according to the average ranks, and the most important N features were selected for the subsequent analysis (the number *N* could be determined by RFE).

Feature selection was performed to keep the most relevant features. Ensemble feature selection can combine the results from different feature selection methods to one major decision, and this can improve the robustness and stability of the final feature selection results (Jong et al., 2004; Abeel et al., 2010; Hoque et al., 2018). Therefore, three different methods were assembled in our feature selection procedure: analysis of variance (ANOVA), random forest, and recursive feature elimination (RFE). The average rank from different methods served as the final feature rank criterion. A schematic overview of our feature selection procedure can be found in Figure 2.

ANOVA was used to compare the features' mean values of IPD group with those of HC groups. The ANOVA *F* value (a measure of significance in *F*-test) was calculated and served as one criterion to rank features: features with a higher *F* value were ranked as more important.

Random forest is a classification method, which trains a series of classifying decision trees by randomly choosing sub-samples of the dataset and sub-features from all the features and then averages the results from different decision trees to avoid overfitting and improve the accuracy. When constructing the decision trees, random forest provides feature importance intrinsically. The feature's importance degree is measured by the impurity proposed by Breiman (2001). Higher impurity implies that the corresponding feature can influence the predictive results more obviously and will be reckoned more important. In our study, we constructed a random forest with 10 decision trees and used the provided importance degree as another criterion for features ranking.

**Figure 3:**
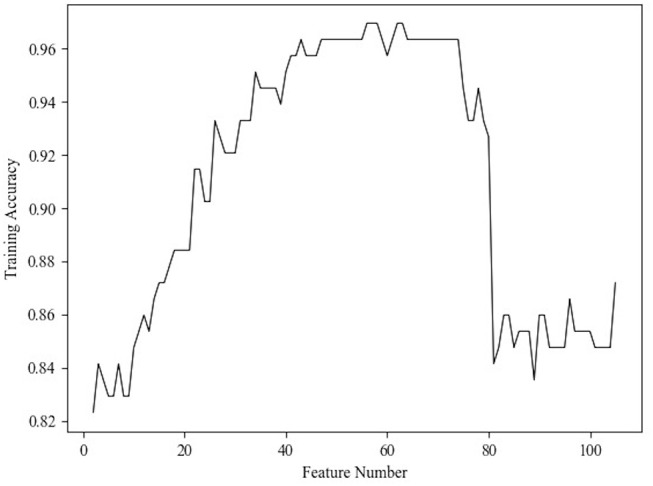
Relationship between the classification accuracy and feature number.

The RFE method selects features by recursively pruning the least important features from current features set, and the estimator in our RFE method was specified as linear support vector machine (SVM). The estimator not only evaluated the importance of features but also gave feedback on training performance of different feature subsets, so that the curve between training accuracy and feature number (Figure 3) could be obtained to determine the optimal number of features. According to the curve, the number was set as 40 in order to reduce the feature dimensionality as much as possible.

Finally, the features were sorted according to the average feature ranks from the above three rankers and the top 40 features were selected for the subsequent classifiers. To illustrate the feature selection results, we would present the top five features selected by each feature selection method in Table 2. All these three methods were implemented on a python package named scikit-learn (Pedregosa et al., 2013). When ranking the features by random forest and RFE, we used all the 164 samples as training set because the validation/test accuracy did not matter when we concentrated on the features' importance in this procedure.

**Table 2:** Results of the ROC curve analyses of the top five features picked by each feature selection method.

	**ANOVA**		**Random Forest**		**RFE**	
	**Features name**	**AUC**	**Features name**	**AUC**	**Features name**	**AUC**
Top1	Minimum	0.83	GryLvNonUni	0.80	DepdEntrp	0.80
Top2	DepdNonUni	0.84	Correlation	0.78	SumEntrp	0.51
Top3	DepdVar	0.81	10 Percentile	0.75	RunEntrp	0.76
Top4	InfMCor1	0.81	Minimum	0.83	ZoneEntrp	0.62
Top5	GryLvNonUni	0.80	RunLthNonUni	0.78	HGLRunEmphs	0.50

### Classification

SVM is one of the most popular machine learning methods and has been used in PD diagnosis (Prashanth et al., 2016; Amoroso et al., 2018; Castillo-Barnes et al., 2018). It tries to find out the optimal hyperplane that minimizes the classification error and maximizes the geometric margin on the training set, which leads to high generalization ability on the new cases (Burges, 1998). In practice, radial basis function (RBF) is usually used as the kernel function to non-linearly map the features to a higher dimensionality and improve the performance of SVM (Han et al., 2012). The RBF kernel function is:

(1) $$\begin{array}{r} K\left(x_{i},x_{j}\right)=\exp\left(-\gamma {∥x_{i}-x_{j}∥}^{2}\right) \end{array}$$

where x_*i*_ and x_*j*_ are the features of samples; γ is a constant parameter.

And the object function of the SVM is:

(2) $$\begin{array}{r} \underset{\omega ,b,\xi }{\min}\frac{1}{2}{∥\omega ∥}^{2}+C\overset{N}{\underset{i=1}{\sum }}\xi_{i}\\ \text{s}.\text{t}.y_{i}\left(\omega x_{i}+b\right)\geq 1-\xi_{i},\;i=1,2,3,\ldots ,N\\ \xi_{i}\geq 0,i=1,2,3,\ldots ,N \end{array}$$

where y_*i*_ is the label of the *i*th sample, and *C* is a constant parameter.

Then, the function can be solved by a Lagrangian function:

(3) $$\begin{array}{l} \underset{\omega ,b,\xi }{\min}\;L\left(\omega ,b,\xi ,\alpha ,\mu \right)=-\frac{1}{2}\overset{N}{\underset{i=1}{\sum }}\overset{N}{\underset{j=1}{\sum }}\alpha_{i}\alpha_{j}y_{i}y_{j}\exp\left(-\gamma ||x_{i}-x_{j}{||}^{2}\right)\\ \;+\overset{N}{\underset{i=1}{\sum }}\alpha_{i} \end{array}$$

At last, the obtained SVM function is:

(4) $$\begin{array}{r} f\left(x\right)=\omega x+b \end{array}$$

There are two parameters (*C*, γ) that can be adjusted according to a specific task and dataset. The parameter *C* is a penalty parameter, which determines the error tolerance when we train an SVM. The parameter γ adjusts the effect of RBF.

The order of the magnitudes of selected features was different; therefore, prior to being fed to the SVM classifier, the features were standardized to zero mean and unit variance. In addition, the standardized features were transformed into an orthogonal space to make the features more discriminating by principal components analysis (PCA) (Groth et al., 2013). Then, these features were input into the SVM classifier. the 3-fold cross-validation with 10 repetitions was used to reduce the influence of dataset partition during the procedure of training and testing. In other words, in each round of the training and testing, we randomly separated the 164 samples into 3-folds. Then, 2-folds (109 samples) were used as training set and the left fold (55 samples) was used as testing set. In our study, the kernel function of SVM was set as RBF conventionally. Parameters *C* and γ were set as 30 and 0.001 by the grid search method.

The performance of the SVM classification was evaluated using the area under the receiver operating characteristic (ROC) curve, accuracy, specificity, and sensitivity.

### Statistical Analysis

Two-tailed unpaired *t*-test was used to compare selected features between HCs and IPDs, and Bonferroni correction was used to correct the error of multiple comparisons. The adjusted *p*-value was set at 0.05/*n*, where *n* = time of comparison. ROC was used to further test the selected features in differentiating IPD from HC *via* the area under ROC (AUC).

Two-tailed Pearson linear correlation was also utilized to study the correlations between the selected features and the UPDRS-III scores of the IPDs. All the above statistical analyses were processed by SPSS Statistics version 25 for mac.

## Results

### Classification Performance

**Figure 4:**
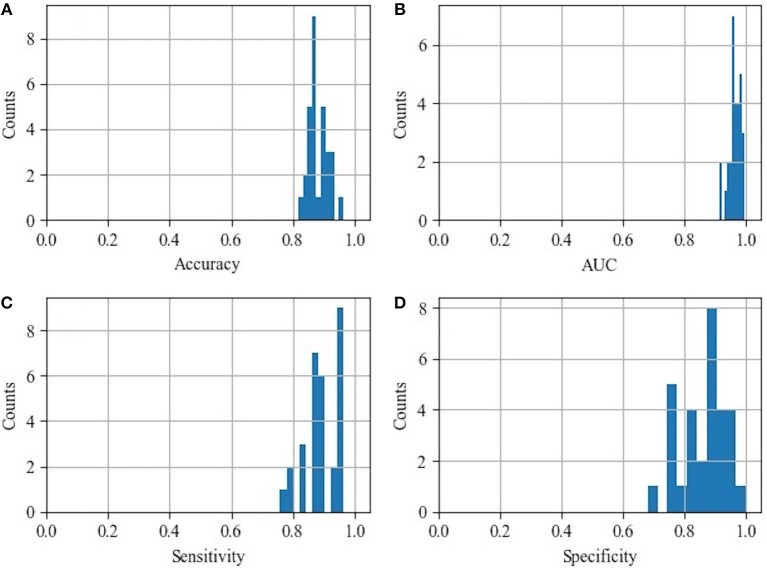
Histograms of the classification performance. **(A)** Histogram of accuracy: 0.88 ± 0.03; **(B)** histogram of AUC: 0.96 ± 0.02; **(C)** histogram of sensitivity: 0.89 ± 0.06; **(D)** histogram of specificity: 0.87 ± 0.07.

**Figure 5:**
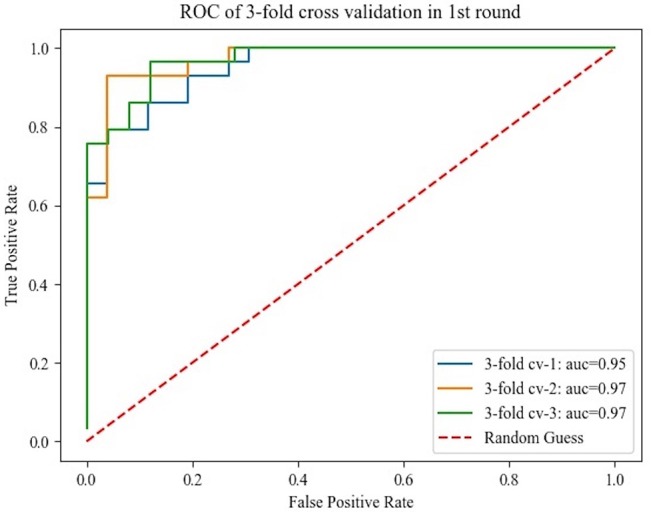
Receiver operating characteristics curve obtained from the 3-fold cross-validation in the first round.

The histograms of the results of the 3-fold cross-validation with 10 repetitions are shown in Figure 4, with the accuracy ranging from 0.80 to 0.95 (centralized at 0.85). The AUC was stable and approximately centralized at 0.95, while the sensitivity and specificity were relatively unstable, ranging from 0.8 to 1.0. Statistically, the AUC, accuracy, sensitivity, and specificity were 0.96 ± 0.02, 0.88 ± 0.03, 0.89 ± 0.06, and 0.87 ± 0.07 (mean ± standard deviation), respectively. To make the performance illustration more concrete and clearer, the cross-validation result in the first round was chosen as a representative example (Table 3; Figure 5). According to the classification performance, the selected features offered informative contents to the SVM and could produce the ROC with average AUC beyond 0.95. As a contrast, the STS was visualized by a radiologist in 67.52% (52/77) of the cases of HC and 9.20% (8/87) of the cases of IPD, respectively. The STS in differentiating HC from IPD was with an accuracy of 79.88%, a sensitivity of 67.53%, and a specificity of 90.80%, which was overall lower than that of the classification.

**Table 3:** Classification performance of 3-fold cross-validation in the first round.

**Index**	**AUC**	**Accuracy**	**Sensitivity**	**Specificity**
Threefold cross-validation 1	0.95	0.85	0.93	0.77
Threefold cross-validation 2	0.97	0.93	0.93	0.92
Threefold cross-validation 3	0.97	0.87	0.86	0.88
Mean ± SD	0.96 ± 0.01	0.88 ± 0.04	0.91 ± 0.04	0.86 ± 0.08

### Top Five and Representative Features Analysis

ROC curve analysis of the top five picked by each feature selection is summarized in Table 2. The AUC of the top five features selected by ANOVA, random forest, and RFE varied from 0.80 to 0.84, 0.75 to 0.83, and 0.50 to 0.80, respectively.

**Figure 6:**
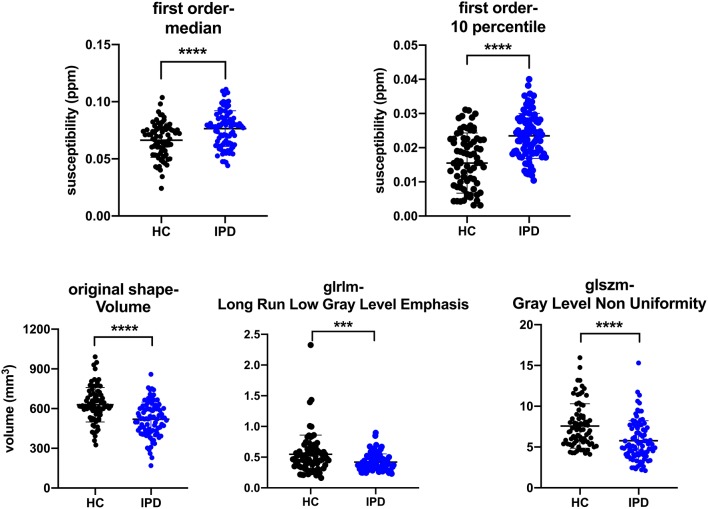
Plots of multiple comparisons of the five representative features between IPD patients and HCs after Bonferroni correction. Dots stand for individual values; horizontal bars stand for mean and standard deviation. ***p ≤ 0.001, ****p < 0.0001 (*a* = 0.05/40).

There were 29 out of the 40 selected features showing significant difference (*p*-value < 0.05/40) between IPD and HC groups, and five features were selected as representative in Table 4 to show the details (all *t*-test results can be found in supplementary S2). The 10th percentile (0.023 ± 0.007 vs. 0.015 ± 0.009, *p* < 0.0001) and median (0.076 ± 0.016 vs. 0.066 ± 0.015, *p* < 0.0001) in IPD patients were higher than those in HCs; while, GLRLM-Long Run Low Gray Level Emphasis (0.420 ± 0.133 vs. 0.546 ± 0.312, *p* = 0.001), GLSZM-Gray Level Non-Uniformity (5.769 ± 2.442, 7.583 ± 2.707, *p* < 0.0001), and volume (519.514 ± 128.743 vs. 629.073 ± 129.558, *p* < 0.0001) in IPD patients were lower than those in HCs. The AUC of these five features varied from 0.64 to 0.75 (Table 4; Figure 6).

**Table 4:** Unpaired *t*-test performances of the five representative features.

**Features Name**	**IPD**	**HC**	***P*-value**	**AUC**
10 th percentile	0.023 ± 0.007	0.015 ± 0.009	1.49E−9	0.75
Median	0.076 ± 0.016	0.066 ± 0.015	4.10E−5	0.68
Volume	519.514 ± 128.743	629.073 ± 129.558	2.46E−7	0.73
LRunLGREmphs	0.420 ± 0.133	0.546 ± 0.312	1.00E−3	0.64
GryLvNonUniS	5.769 ± 2.442	7.583 ± 2.707	1.40E−5	0.71

*LRunLGREmphs, GLRLM-Long Run Low Gray Level Emphasis; GryLvNonUniS, GLSZM-Gray Level Non-Uniformity; the units of 10th Percentile and Median were parts per million (ppm); the unit of volume was mm^3^; there were no units of the remaining two features*.

The correlation between the five features and the UPDRS-III score of IPD was as follows: 10th percentile of the first order was positively correlated with UPDRS-III score (*r* = 0.35, *p* = 0.001); median of the first order, GLSZM-Gray Level Non-Uniformity, GLRLM-Long Run Low Gray Level Emphasis, and volume were not correlated with UPDRS-III score (*r* = 0.15, −0.02, −0.17, and −0.05 and *p* = 0.19, 0.87, 0.12, and 0.65, respectively).

## Discussion

Since iron accumulation in the SN plays a vital role in the progression of PD, visualization and quantification of increased iron are thought to have the potential to assist in the diagnosis and evaluation on PD (He et al., 2015; Noh et al., 2015; Reiter et al., 2015). Howerver, iron deposition is spatially heterogeneous and the nigrosome territory may individually vary (Schmidt et al., 2017). Single iron-sensitive nigrosome imaging for visualization or simple utilization of the mean of susceptibility might be subjective, inssufficient, and inaccurate. Radiomics based on QSM could offer the means to overcome these deficiencies. Our preliminary results verified that radiomic features of the nigrosome-1 region of the SN were different between IPD patients and HCs. The performance of the SVM approach based on these radiomic features was better than that of the radiologist in our study, especially on accuracy and sensitivity, and was similar to that of a meta-analysis based on visualizing the STS at 3.0 T (Mahlknecht et al., 2017). The reason on one hand might be the limitation of radiologists' visual discernment on the sign especially between the early stage IPD and the HCs, and on the other hand, more HCs in our group showed loss of STS unilaterally or bilaterally as a result of individual nigrosome-1 variants or potential preclinical PD, which might lead to a similar perfromance to the previous visual assessment investigations.

As the first-order features describe the distribution of the voxel intensities within a VOI, they might have the potential to reflect the iron deposition spatially. Our study found that the representive features of the 10th percentile and median in the nigrosome-1 region of the SN in IPD patients were bigger than those of the HCs, and the 10th percentile positively correlated with the UPDRS-III, suggesting that the 10th percentile and median of intra-voxel susceptibility (iron content) might better differentiate IPD from HC and the 10th percentile might be a biomarker for the progression of the IPD. The 10th percentile and median represent the first 10 and 50% proprotion of voxels with positive order of susceptibility, respetively. The voxels with lower susceptibility can partially seperate the overlap of voxels between IPD and HC (Figure 6, first row) and might outperform the mean value, which is similar to the findings of a histogram analytical study that using the proportion of voxels with susceptibility lower than 70 ppb as the threshold could better differentiate early IPD from HC compared to the other thresholds including mean value (Kim et al., 2018). The volume, one of the shape-based features, was found smaller in IPD than that in HC, which is consistent with previous studies (Sasaki et al., 2006; Minati et al., 2007; Menke et al., 2009; Ziegler et al., 2013), suggesting that the iron deposition increases and dopaminergic neurons lose in IPD (Duguid et al., 1986). The second-order features describe the relationship of all voxel pairs within a segamented VOI. GLRLM-Long Run Low Gray Level Emphasis and GLSZM-Gray Level Non-Uniformity describe the joint distribution of long run lengths with lower gray-level values and the similarity of the gray-level intensity values, respectively, which were both found lower in the IPD patients compared to those in HCs in our study. This reflected the fact that iron accumulation of the nigrosome-1-containing part of SN was spatially more uniform in IPD, which was consistent with the loss of STS in IPD. Therefore, radiomic features could be an objective means to assess iron accumulation and neuromelanin loss in SN spatially and could be a promising time-saving tool in assisting in the diagnosis of IPD.

Furthermore, apart from analyzing the selected radiomic features in this study, we also trained SVM to classify the subjects as IPD patients or HCs automatically. Recently, several studies have proved that it is effective to diagnose PD using SVM and different features (Prashanth et al., 2016; Amoroso et al., 2018; Castillo-Barnes et al., 2018) with an accuracy ranging from 0.70 to 0.96. However, few of them trained the SVM using the radiomic features as input. In our study, we found that the radiomic featues also provided discraminative information to the SVM classifier and could reach an accuracy of 0.88. The SVM classifier could ultilize high-dimensional features automatically, but its results intrinsically fluctuate according to different training datasets and testing datesets (Figure 4).

There are several limitations to this study. First, only the nigrosome-1 territory was segmented, which might bias the results. It is known that the nigrosome-1 is the first to be involved in the progression of PD (Sung et al., 2018). Since most of our IPD cases were at the the early stages (68 out of 77 cases were at Hoehn and Yahr stage 1–2), we expect that less bias was introduced. Second, instead of defining some specific features that might better characterize the nigrosome-1 pathological changes and subsequently well-differentiate the IPD from HCs, only 105 common radiomic features were extracted in the study, which might be insufficient. Third, we only used SVM as the classifier, rather than comparing or assembling the performances of different machine learning methods. In the future, we can improve the accuracy by exploring novel features and classifiers.

In conclusion, radiomic features of the SN based on QSM could be useful in the diagnosis of IPD and could serve as a surrogate marker for the qualitiative (visualization) and conventional quantitative evalaution of STS or nigrosome-1.

## Ethics Statement

This study was approved by Review Board of Ruijin Hospital, Shanghai Jiaotong University and written informed consent was given and signed by all the participants.

## Author Contributions

ZC and FY designed the study. NH, HX, ZJ, RT, and YL collected the MRI data. ZC and JZ segmented the QSM data. JZ, YW, and DQ analyzed the data and interpreted the radiomic features. ZC and JZ wrote the manuscript draft. EH revised the manuscript.

### Conflict of Interest Statement

The authors declare that the research was conducted in the absence of any commercial or financial relationships that could be construed as a potential conflict of interest.
