# Supplementary material for: Radiomic Features of the Nigrosome-1 Region of the Substantia Nigra: Using Quantitative Susceptibility Mapping to Assist the Diagnosis of Idiopathic Parkinson's Disease
Source: Front Aging Neurosci. 2019 Jul 16;11:167. doi: 10.3389/fnagi.2019.00167 (PMC6648885; doi:10.3389/fnagi.2019.00167)
Supplement: Supplementary file 1 [file Table_1.docx]

**Table S1-1 Description and equation of the first-order features**

| **Index** | **Features name** | **Description** | **Equation** |
| --- | --- | --- | --- |
| 1 | Minimum | the minimum susceptibility in the VOI | min(X) |
| 2 | 10Percentile | the 10^th^ percentile of the sorted susceptibility in the VOI | 10th_percentile(X) |
| 3 | Kurtosis | the ‘peakedness’ of the susceptibility in the VOI, A higher kurtosis implies that the mass of the distribution is concentrated towards the tails rather than towards the mean | $kurtosis=\frac{\mu_{4}}{\sigma^{4}}=\frac{\frac{1}{N_{p}}\sum_{i=1}^{N_{p}} (\mathbf{X}(i)-\bar{X})^{4}}{\left(\frac{1}{N_{p}}\sum_{i=1}^{N_{p}} (\mathbf{X}(i)-\bar{X})^{2}\right)^{2}}$ |
| 4 | Skewness | asymmetry of the distribution of the susceptibility about the Mean value | $skewness=\frac{\mu_{3}}{\sigma^{3}}=\frac{\frac{1}{N_{p}}\sum_{i=1}^{N_{p}} (\mathbf{X}(i)-\bar{X})^{3}}{\left(\sqrt{\frac{1}{N_{p}}\sum_{i=1}^{N_{p}} (\mathbf{X}(i)-\bar{X})^{2}}\right)^{3}}$ |
| 5 | RootMeanSquared  (RMS) | square-root of the mean of all the squared intensity values | $RMS=\sqrt{\frac{1}{N_{p}}\sum_{i=1}^{N_{p}} (\mathbf{X}(i))^{2}}$ |
| 6 | Median | median of the sorted susceptibility in the VOI | Median(X) |
| 7 | Entropy | randomness of the susceptibility | $entropy=-\sum_{i=1}^{N_{g}} p(i)\log_{2}p(i)$ |
| 8 | Energy | the magnitude of susceptibility in the VOI | $energy=\sum_{i=1}^{N_{p}} (\mathbf{X}(i))^{2}$ |
| 9 | TotalEnergy | Energy feature scaled by the volume of the voxel in VOI | $total\_energy=V_{voxel}\sum_{i=1}^{N_{p}} (\mathbf{X}(i))^{2}$ |
| 10 | 90Percentile | the 90^th^ percentile of the sorted susceptibility in the VOI | 90th_percentile(X) |
| **Index** | **Features name** | **Description** | **Equation** |
| 11 | Mean | the average susceptibility within the VOI. | $mean=\frac{1}{N_{p}}\sum_{i=1}^{N_{p}} \mathbf{X}(i)$ |
| 12 | MeanAbsolute  Deviation (MAD) | the mean distance of all susceptibilities from the Mean Value in the VOI | $MAD=\frac{1}{N_{p}}\sum_{i=1}^{N_{p}} \vert\mathbf{X}(i)-\bar{X}\vert$ |
| 13 | RobustMeanAbsolute  Deviation (RMAD) | the mean distance of all intensity values from the Mean Value calculated between 10^th^ and 90^th^ percentile. | $rMAD=\frac{1}{N_{10-90}}\sum_{i=1}^{N_{10-90}} \vert\mathbf{X}_{10-90}(i)-\bar{X}_{10-90}\vert$ |
| 14 | Range | The range of susceptibility in the VOI | $range=\max(\mathbf{X})-\min(\mathbf{X})$ |
| 15 | InterquartileRange | the difference between 25^th^ and 75^th^ percentile within the VOI | $interquartile\_range=\mathbf{P}_{75}-\mathbf{P}_{25}$ |
| 16 | Maximum | the maximum susceptibility in the VOI | max(X) |
| 17 | Variance | the amount of dispersion from the Mean Value | $variance=\frac{1}{N_{p}}\sum_{i=1}^{N_{p}} (\mathbf{X}(i)-\bar{X})^{2}$ |
| 18 | Uniformity | the homogeneity of the image | $uniformity=\sum_{i=1}^{N_{g}} p(i)^{2}$ |

The first-order features describe how the individual susceptibilities distribute statistically.

*X* is a set of *Np* voxels included in the VOI; *P(i)* is the first order histogram with *Ng* discrete intensity levels.

**Table S1-2 Description and equation of the texture features based on gray level co-occurrence matrices (GLCM)**

| **Index** | **Features name** | **Description** | **Equation** |
| --- | --- | --- | --- |
| 1 | Correlation | the image complexity. the linear dependency of gray level values to their respective voxels in the GLCM | $correlation=\frac{\sum_{i=1}^{N_{g}} \sum_{j=1}^{N_{g}} p(i,j)ij-\mu_{x}\mu_{y}}{\sigma_{x}(i)\sigma_{y}(j)}$ |
| 2 | Informational Measure of Correlation 1 (IMC1) | the complexity of the texture | $IMC1=\frac{HXY-HXY1}{\max\{HX,HY\}}$ |
| 3 | Inverse Difference Normalized (IDN) | the local homogeneity of an image | $IDN=\sum_{k=0}^{N_{g}-1} \frac{p_{x-y}(k)}{1+\left(\frac{k}{N_{g}}\right)}$ |
| 4 | Informational Measure of Correlation 2 (IMC2) | the correlation between the probability distributions of i and j | $IMC2=\sqrt{1-e^{-2(HXY2-HXY)}}$ |
| 5 | DifferenceVariance | the heterogeneity that places higher weights on differing intensity level pairs that deviate more from the mean | $difference\_variance=\sum_{k=0}^{N_{g}-1} (k-DA)^{2}p_{x-y}(k)$ |
| 6 | InverseVariance | a measure of the local homogeneity in an image | $inverse\_variance=\sum_{k=1}^{N_{g}-1} \frac{p_{x-y}(k)}{k^{2}}$ |
| 7 | DifferenceEntropy | the randomness in neighborhood intensity value differences | $difference\_entropy=\sum_{k=0}^{N_{g}-1} p_{x-y}(k)\log_{2}(p_{x-y}(k))$ |
| 8 | Contrast | the local intensity variation | $contrast=\sum_{i=1}^{N_{g}} \sum_{j=1}^{N_{g}} (i-j)^{2}p(i,j)$ |
| 9 | MaximumProbability | occurrences of the most predominant pair of neighboring intensity values | $maximum\_probability=\max(p(i,j))$ |
| **Index** | **Features name** | **Description** | **Equation** |
| 10 | SumEntropy | the sum of neighborhood intensity value differences | $sum\_entropy=\sum_{k=2}^{2N_{g}} p_{x+y}(k)\log_{2}(p_{x+y}(k))$ |
| 11 | Inverse Difference Moment (IDM) | the local homogeneity of an image | $IDM=\sum_{k=0}^{N_{g}-1} \frac{p_{x-y}(k)}{1+k^{2}}$ |
| 12 | DifferenceAverage | the relationship between occurrences of pairs with similar intensity values and occurrences of pairs with differing intensity values | $difference\_average=\sum_{k=0}^{N_{g}-1} kp_{x-y}(k)$ |
| 13 | Autocorrelation | the magnitude of the fineness and coarseness of texture | $autocorrelation=\sum_{i=1}^{N_{g}} \sum_{j=1}^{N_{g}} p(i,j)ij$ |
| 14 | JointAverage | the mean gray level intensity of the intensity distribution | $joint\_average=\sum_{i=1}^{N_{g}} \sum_{j=1}^{N_{g}} p(i,j)i$ |
| 15 | SumAverage | the relationship between occurrences of pairs with lower intensity values and occurrences of pairs with higher intensity values | $sum\_average=\sum_{k=2}^{2N_{g}} p_{x+y}(k)k$ |
| 16 | SumSquares | a measure in the distribution of neigboring intensity level pairs about the mean intensity level in the GLCM | $sum\_squares=\sum_{i=1}^{N_{g}} \sum_{j=1}^{N_{g}} (i-\mu_{x})^{2}p(i,j)$ |
| 17 | ClusterShade | a measure of the skewness and uniformity of the GLCM | $cluster\_shade=\sum_{i=1}^{N_{g}} \sum_{j=1}^{N_{g}} (i+j-\mu_{x}-\mu_{y})^{3}p(i,j)$ |
| **Index** | **Features name** | **Description** | **Equation** |
| 18 | ClusterProminence | the skewness of the GLCM | $cluster\_prominence=\sum_{i=1}^{N_{g}} \sum_{j=1}^{N_{g}} (i+j-\mu_{x}-\mu_{y})^{4}p(i,j)$ |
| 19 | JointEnergy | homogeneous patterns in the image | $joint\_energy=\sum_{i=1}^{N_{g}} \sum_{j=1}^{N_{g}} (p(i,j))^{2}$ |
| 20 | JointEntropy | the randomness in neighborhood intensity values | $joint\_entropy=-\sum_{i=1}^{N_{g}} \sum_{j=1}^{N_{g}} p(i,j)\log_{2}(p(i,j))$ |
| 21 | Inverse Difference (ID) | the local homogeneity of an image | $ID=\sum_{k=0}^{N_{g}-1} \frac{p_{x-y}(k)}{1+\left(\frac{k}{N_{g}}\right)}$ |
| 22 | Inverse Difference Moment Normalized (IDMN) | the normalized IDM, a measure of the local homogeneity of an image | $IDMN=\sum_{k=0}^{N_{g}-1} \frac{p_{x-y}(k)}{1+\left(\frac{k^{2}}{N_{g}^{2}}\right)}$ |
| 23 | ClusterTendency | a measure of groupings of voxels with similar gray-level values | $cluster\_tendency=\sum_{i=1}^{N_{g}} \sum_{j=1}^{N_{g}} (i+j-\mu_{x}-\mu_{y})^{2}p(i,j)$ |

The (i,j)^th^ element of GLCM represents the frequency of the combination of levels *i* and *j* of two pixels occurring in the image, which are separated by a distance of *δ* pixels along angle *θ.*

*p(i,j)* is the normalized GLCM; *p_x_(i)*, *p_y_(i)* are the marginal row and column probability respectively; *μ_x_, μ_y_* and *σ_x_, σ_y_* denote the mean and standard deviations of the row and column sums of the GLCM respectively; HX and HY are the entropy of *p_x_* and *p_y_* respectively; HXY is be the entropy of *p(i,j).*

$p_{x+y}(k)=\sum_{i=1}^{N_{g}} \sum_{j=1}^{N_{g}} p(i,j),\text{ where }i+j=k,\text{ and }k=2,3,\ldots,2N_{g}$; $p_{x-y}(k)=\sum_{i=1}^{N_{g}} \sum_{j=1}^{N_{g}} p(i,j),\text{ where }|i-j|=k,\text{ and }k=0,1,\ldots,N_{g}-1$

**Table S1-3 Description and equation of the texture features based on Gray Level Dependence Matrix (GLDM)**

| **Index** | **Features name** | **Description** | **Equation** |
| --- | --- | --- | --- |
| 1 | DependenceEntropy | the randomness of GLDM. bigger Dependence Entropy implies more complex textural | $DE=-\sum_{i=1}^{N_{g}} \sum_{j=1}^{N_{d}} p(i,j)\log_{2}(p(i,j))$ |
| 2 | DependenceVariance | the variance in dependence size in the image | $DV=\sum_{i=1}^{N_{g}} \sum_{j=1}^{N_{d}} p(i,j)(j-\mu)^{2}\text{, where}\mu=\sum_{i=1}^{N_{g}} \sum_{j=1}^{N_{d}} jp(i,j)$ |
| 3 | GrayLevelNonUniformity  (GLN) | the similarity of gray-level intensity values in the image, where a lower GLN value correlates with a greater similarity in intensity values | $GLN=\frac{\sum_{i=1}^{N_{g}} \left(\sum_{j=1}^{N_{d}} \mathbf{P}(i,j)\right)^{2}}{N_{z}}$ |
| 4 | LargeDependenceEmphasis  (LDE) | the distribution of large dependencies, with a greater value indicative of larger dependence and more homogeneous textures | $LDE=\frac{\sum_{i=1}^{N_{g}} \sum_{j=1}^{N_{d}} \mathbf{P}(i,j)j^{2}}{N_{z}}$ |
| 5 | DependenceNonUniformity  Normalized(DNN) | the similarity of dependence throughout the image, with a lower value indicating more homogeneity among dependencies in the image. | $DNN=\frac{\sum_{j=1}^{N_{d}} \left(\sum_{i=1}^{N_{g}} \mathbf{P}(i,j)\right)^{2}}{N_{z}^{2}}$ |
| 6 | LargeDependenceLowGray  LevelEmphasis(LDLGLE) | the joint distribution of large dependence with lower gray-level values | $LDLGLE=\frac{\sum_{i=1}^{N_{g}} \sum_{j=1}^{N_{d}} \frac{\mathbf{P}(i,j)j^{2}}{i^{2}}}{N_{z}}$ |
| 7 | LowGrayLevelEmphasis  (LGLE) | the distribution of low gray-level values, with a higher value indicating a greater concentration of low gray-level values in the image | $LGLE=\frac{\sum_{i=1}^{N_{g}} \sum_{j=1}^{N_{d}} \frac{\mathbf{P}(i,j)}{i^{2}}}{N_{z}}$ |
| 8 | DependenceNonUniformity  (DN) | the similarity of dependence throughout the image, with a lower value indicating more homogeneity among dependencies in the image | $DN=\frac{\sum_{j=1}^{N_{d}} \left(\sum_{i=1}^{N_{g}} \mathbf{P}(i,j)\right)^{2}}{N_{z}}$ |
| **Index** | **Features name** | **Description** | **Equation** |
| 9 | SmallDependenceHighGray  LevelEmphasis(SDHGLE) | the joint distribution of small dependence with higher gray-level values | $SDHGLE=\frac{\sum_{i=1}^{N_{g}} \sum_{j=1}^{N_{d}} \frac{\mathbf{P}(i,j)i^{2}}{j^{2}}}{N_{z}}$ |
| 10 | SmallDependenceLowGray  LevelEmphasis(SDLGLE) | the joint distribution of small dependence with lower gray-level values | $SDLGLE=\frac{\sum_{i=1}^{N_{g}} \sum_{j=1}^{N_{d}} \frac{\mathbf{P}(i,j)}{i^{2}j^{2}}}{N_{z}}$ |
| 11 | SmallDependenceEmphasis  (SDE) | the distribution of small dependencies, with a greater value indicative of smaller dependence and less homogeneous textures | $SDE=\frac{\sum_{i=1}^{N_{g}} \sum_{j=1}^{N_{d}} \frac{\mathbf{P}(i,j)}{i^{2}}}{N_{z}}$ |
| 12 | LargeDependenceHighGray  LevelEmphasis(LDHGLE) | the joint distribution of large dependence with higher gray-level values | $LDHGLE=\frac{\sum_{i=1}^{N_{g}} \sum_{j=1}^{N_{d}} \mathbf{P}(i,j)i^{2}j^{2}}{N_{z}}$ |
| 13 | GrayLevelVariance  (GLV) | the variance in grey level in the image | $GLV=\sum_{i=1}^{N_{g}} \sum_{j=1}^{N_{d}} p(i,j)(i-\mu)^{2}\text{, where}\mu=\sum_{i=1}^{N_{g}} \sum_{j=1}^{N_{d}} ip(i,j)$ |
| 14 | HighGrayLevelEmphasis  (HGLE) | the distribution of the higher gray-level values, with a higher value indicating a greater concentration of high gray-level values in the image | $HGLE=\frac{\sum_{i=1}^{N_{g}} \sum_{j=1}^{N_{d}} \mathbf{P}(i,j)i^{2}}{N_{z}}$ |

GLDM quantifies gray level dependencies in an image. A gray level dependency is defined as the number of connected voxels within distance *δ* that are dependent on the center voxel.

*N_g_* is the number of discreet intensity values in the image; *N_d_* is the number of discreet dependency sizes in the image; *N_z_* is the number of dependency zones in the image.

**Table S1-4 Description and equation of the texture features based on Gray Level Run Length Matrix (GLRLM)**

| **Index** | **Features name** | **Description** | **Equation** |
| --- | --- | --- | --- |
| 1 | RunEntropy  (RE) | the randomness of the distribution of run lengths and gray levels. A higher value indicates more heterogeneity in the texture patterns | $RE=-\sum_{i=1}^{N_{g}} \sum_{j=1}^{N_{r}} p(i,j)\log_{2}(p(i,j))$ |
| 2 | LongRunEmphasis  (LRE) | the distribution of long run lengths, with a greater value indicative of longer run lengths and more coarse  structural textures | $LRE=\frac{\sum_{i=1}^{N_{g}} \sum_{j=1}^{N_{r}} \mathbf{P}(i,j)j^{2}}{N_{r}}$ |
| 3 | RunLengthNonUniformity  Normalized(RLNN) | the similarity of run lengths throughout the image, with a lower value indicating more homogeneity among run lengths  in the image | $RLNN=\frac{\sum_{j=1}^{N_{r}} \left(\sum_{i=1}^{N_{g}} \mathbf{P}(i,j)\right)^{2}}{N_{r}^{2}}$ |
| 4 | RunVariance  (RV) | the variance in runs for the run lengths | $\begin{matrix} RV=\sum_{i=1}^{N_{g}} \sum_{j=1}^{N_{r}} p(i,j)(j-\mu)^{2},\text{ } \\ \text{where }\mu=\sum_{i=1}^{N_{g}} \sum_{j=1}^{N_{r}} p(i,j)j \end{matrix}$ |
| 5 | GrayLevelNonUniformity  (GLN) | the similarity of gray-level intensity values in the image, a lower GLN value correlates with a greater similarity  in intensity values | $GLN=\frac{\sum_{i=1}^{N_{g}} \left(\sum_{j=1}^{N_{d}} \mathbf{P}(i,j)\right)^{2}}{N_{z}}$ |
| 6 | LongRunLowGrayLevel  Emphasis(LRLGLE) | the joint distribution of long run lengths with lower  gray-level values | $LRLGLE=\frac{\sum_{i=1}^{N_{g}} \sum_{j=1}^{N_{r}} \frac{\mathbf{P}(i,j)j^{2}}{i^{2}}}{N_{r}}$ |
| **Index** | **Features name** | **Description** | **Equation** |
| 7 | ShortRunLowGrayLevelEmphasis  (SRLGLE) | measures the joint distribution of shorter run lengths with lower gray-level values | $SRLGLE=\frac{\sum_{i=1}^{N_{g}} \sum_{j=1}^{N_{r}} \frac{\mathbf{P}(i,j)}{i^{2}j^{2}}}{N_{r}}$ |
| 8 | ShortRunEmphasis  (SRE) | the distribution of short run lengths, a greater value indicates shorter run lengths and more fine textural textures | $SRE=\frac{\sum_{i=1}^{N_{g}} \sum_{j=1}^{N_{r}} \frac{\mathbf{P}(i,j)}{j^{2}}}{N_{r}}$ |
| 9 | LowGrayLevelRunEmphasis  (LGLRE) | the distribution of low gray-level values, with a higher value indicating a greater concentration of low gray-level values  in the image | $LGLRE=\frac{\sum_{i=1}^{N_{g}} \sum_{j=1}^{N_{r}} \frac{\mathbf{P}(i,j)}{i^{2}}}{N_{r}}$ |
| 10 | RunLengthNonUniformity  (RLN) | the similarity of run lengths throughout the image, with a lower value indicating more homogeneity among run lengths  in the image | $RLN=\frac{\sum_{j=1}^{N_{r}} \left(\sum_{i=1}^{N_{g}} \mathbf{P}(i,j)\right)^{2}}{N_{r}}$ |
| 11 | RunPercentage  (RP) | the coarseness of the texture by considering the ratio of number of runs and number of voxels in the VOI | $RP=\frac{N_{r}}{N_{p}}$ |
| 12 | LongRunHighGrayLevel  Emphasis(LRHGLE) | the joint distribution of long run lengths with higher  gray-level values | $LRHGLE=\frac{\sum_{i=1}^{N_{g}} \sum_{j=1}^{N_{r}} \mathbf{P}(i,j)i^{2}j^{2}}{N_{r}}$ |
| 13 | HighGrayLevelRun  Emphasis (HGLRE) | the distribution of the higher gray-level values, with a higher value indicating a greater concentration of high gray-level values | $HGLRE=\frac{\sum_{i=1}^{N_{g}} \sum_{j=1}^{N_{r}} \mathbf{P}(i,j)i^{2}}{N_{r}}$ |
| **Index** | **Features name** | **Description** | **Equation** |
| 14 | ShortRunHighGrayLevel  Emphasis(SRHGLE) | the joint distribution of shorter run lengths with  higher gray-level values | $SRHGLE=\frac{\sum_{i=1}^{N_{g}} \sum_{j=1}^{N_{r}} \frac{\mathbf{P}(i,j)i^{2}}{j^{2}}}{N_{r}}$ |
| 15 | GrayLevelVariance  (GLV) | the variance in grey level in the image | $\begin{matrix} GLV=\sum_{i=1}^{N_{g}} \sum_{j=1}^{N_{d}} p(i,j)(i-\mu)^{2}\text{, } \\ \text{where }\mu=\sum_{i=1}^{N_{g}} \sum_{j=1}^{N_{d}} ip(i,j) \end{matrix}$ |
| 16 | GrayLevelNonUniformity  Normalized (GLNN) | the homogeneity of the image array, a greater uniformity implies a greater homogeneity or a smaller range of discrete intensity values | $GLNN=\frac{\sum_{i=1}^{N_{g}} \left(\sum_{j=1}^{N_{d}} \mathbf{P}(i,j)\right)^{2}}{\sum_{i=1}^{N_{g}} \sum_{j=1}^{N_{d}} \mathbf{P}(i,j)^{2}}$ |

GLRLM quantifies gray level runs, which are defined as the length in number of pixels, of consecutive pixels that have the same gray level value. In a gray level run length matrix *P(i,j)*, the (i,j)th element describes the number of runs with gray level *i* and length *j* along angle *θ*

*p(i,j)* be the normalized run length matrix; *Ng* be the number of discreet intensity values in the image; *Nr* be the number of discreet run lengths in the image; *Np* be the number of voxels in the image.

**Table S1-5 Description and equation of the texture features based on Gray Level Size Zone Matrix (GLSZM)**

| **Index** | **Features name** | **Description** | **Equation** |
| --- | --- | --- | --- |
| 1 | ZoneEntropy  (ZE) | the randomness in the distribution of zone sizes and gray levels. A higher value indicates more heterogeneity in the texture patterns | $ZE=-\sum_{i=1}^{N_{g}} \sum_{j=1}^{N_{s}} p(i,j)\log_{2}(p(i,j))$ |
| 2 | GrayLevelNonUniformity  (GLN) | the variability of gray-level intensity values in the image, with a lower value indicating more homogeneity in intensity values | $GLN=\frac{\sum_{i=1}^{N_{g}} \left(\sum_{j=1}^{N_{s}} \mathbf{P}(i,j)\right)^{2}}{N_{z}}$ |
| 3 | GrayLevelNonUniformity  Normalized(GLNN) | the variability of gray-level intensity values in the image, with a lower value indicating a greater similarity in intensity values.The normalized version of the GLN | $GLNN=\frac{\sum_{i=1}^{N_{g}} \left(\sum_{j=1}^{N_{s}} \mathbf{P}(i,j)\right)^{2}}{N_{z}^{2}}$ |
| 4 | SmallAreaEmphasis  (SAE) | the distribution of small size zones, with a greater value indicative of more smaller size zones and more fine textures | $SAE=\frac{\sum_{i=1}^{N_{g}} \sum_{j=1}^{N_{s}} \frac{\mathbf{P}(i,j)}{j^{2}}}{N_{z}}$ |
| 5 | SizeZoneNonUniformity  (SZN) | the variability of size zone volumes in the image, with a lower value indicating more homogeneity in  size zone volumes | $SZN=\frac{\sum_{j=1}^{N_{s}} \left(\sum_{i=1}^{N_{g}} \mathbf{P}(i,j)\right)^{2}}{N_{z}}$ |
| 6 | SizeZoneNonUniformity  Normalized (SZNN) | the variability of size zone volumes throughout the image, with a lower value indicating more homogeneity among zone size volumes in the image, the normalized  version of the SZN | $SZNN=\frac{\sum_{j=1}^{N_{s}} \left(\sum_{i=1}^{N_{g}} \mathbf{P}(i,j)\right)^{2}}{N_{z}^{2}}$ |
| **Index** | **Features name** | **Description** | **Equation** |
| 7 | SmallAreaLowGrayLevelEmphasis(SALGLE) | the proportion in the image of the joint distribution of smaller size zones with lower gray-level values | $SALGLE=\frac{\sum_{i=1}^{N_{g}} \sum_{j=1}^{N_{s}} \frac{\mathbf{P}(i,j)}{i^{2}j^{2}}}{N_{z}}$ |
| 8 | LargeAreaHighGrayLevelEmphasis(LAHGLE) | the proportion in the image of the joint distribution of larger size zones with higher gray-level values | $LAHGLE=\frac{\sum_{i=1}^{N_{g}} \sum_{j=1}^{N_{s}} \mathbf{P}(i,j)i^{2}j^{2}}{N_{z}}$ |
| 9 | ZoneVariance(ZV) | the variance in zone size volumes for the zones | $ZV=\sum_{i=1}^{N_{g}} \sum_{j=1}^{N_{s}} p(i,j)(j-\mu)^{2}\text{ where }\mu=\sum_{i=1}^{N_{g}} \sum_{j=1}^{N_{s}} p(i,j)j$ |
| 10 | LowGrayLevelZoneEmphasis(LGLZE) | the distribution of lower gray-level size zones, with a higher value indicating a greater proportion of lower gray-level values and size zones in the image | $LGLZE=\frac{\sum_{i=1}^{N_{g}} \sum_{j=1}^{N_{s}} \frac{\mathbf{P}(i,j)}{i^{2}}}{N_{z}}$ |
| 11 | GrayLevelVariance(GLV) | the variance in gray level intensities for the zones | $GLV=\sum_{i=1}^{N_{g}} \sum_{j=1}^{N_{s}} p(i,j)(i-\mu)^{2}\text{ where }\mu=\sum_{i=1}^{N_{g}} \sum_{j=1}^{N_{s}} p(i,j)i$ |
| 12 | HighGrayLevelZoneEmphasis (HGLZE) | the distribution of the higher gray-level values, with a higher value indicating a greater proportion of higher gray-level values and size zones in the image | $HGLZE=\frac{\sum_{i=1}^{N_{g}} \sum_{j=1}^{N_{s}} \mathbf{P}(i,j)i^{2}}{N_{z}}$ |
| 13 | LargeAreaEmphasis (LAE) | the distribution of large area size zones, with a greater value indicative of more larger size zones and more coarse textures | $LAE=\frac{\sum_{i=1}^{N_{g}} \sum_{j=1}^{N_{s}} \mathbf{P}(i,j)j^{2}}{N_{z}}$ |
| 14 | LargeAreaLowGrayLevelEmphasis (LALGLE) | the proportion in the image of the joint distribution of larger size zones with lower gray-level values | $LALGLE=\frac{\sum_{i=1}^{N_{g}} \sum_{j=1}^{N_{s}} \frac{\mathbf{P}(i,j)j^{2}}{i^{2}}}{N_{z}}$ |
| **Index** | **Features name** | **Description** | **Equation** |
| 15 | SmallAreaHighGrayLevelEmphasis (SAHGLE) | the proportion in the image of the joint distribution of smaller size zones with higher gray-level values | $SAHGLE=\frac{\sum_{i=1}^{N_{g}} \sum_{j=1}^{N_{s}} \frac{\mathbf{P}(i,j)i^{2}}{j^{2}}}{N_{z}}$ |
| 16 | ZonePercentage(ZP) | the coarseness of the texture by taking the ratio of number of zones and number of voxels in the VOI | $ZP=\frac{N_{z}}{N_{p}}$ |

GLSZM quantifies gray level zones in an image. A gray level zone is defined as the number of connected voxels that share the same gray level intensity. In a gray level size zone matrix P(i,j), the (i,j)th element equals the number of zones with gray level *i* and size *j* appear in image.

*p(i,j)* is the normalized size zone matrix; *Ng* be the number of discreet intensity values in the image; *Ns* be the number of discreet zone sizes in the image; *Np* be the number of voxels in the image; *Nz* be the number of zones.

**Table S1-6 Description and equation of the texture features based on Neighbouring Gray Tone Difference Matrix (NGTDM)**

| **Index** | **Features name** | **Description** | **Equation** |
| --- | --- | --- | --- |
| 1 | Complexity | bigger value means that the image is non-uniform and there are many rapid changes in gray level intensity | $Complexity=\frac{1}{N_{v,p}}\sum_{i=1}^{N_{g}} \sum_{j=1}^{N_{g}} \vert i-j\vert\frac{p_{i}s_{i}+p_{j}s_{j}}{p_{i}+p_{j}}\text{, where }p_{i}\neq0,p_{j}\neq0$ |
| 2 | Busyness | A high value for busyness indicates the image is with rapid changes of intensity between pixels and its neighbourhood | $Busyness=\frac{\sum_{i=1}^{N_{g}} p_{i}s_{i}}{\sum_{i=1}^{N_{g}} \sum_{j=1}^{N_{g}} \vert ip_{i}-jp_{j}\vert}\text{, where }p_{i}\neq0,p_{j}\neq0$ |
| 3 | Contrast | high value means that an image is with a large range of gray levels, with large changes between voxels and their neighbourhood | $\begin{matrix} Contrast=\left(\frac{1}{N_{g,p}(N_{g,p}-1)}\sum_{i=1}^{N_{g}} \sum_{j=1}^{N_{g}} p_{i}p_{j}(i-j)^{2}\right)\left(\frac{1}{N_{v,p}}\sum_{i=1}^{N_{g}} s_{i}\right)\text{, } \\ \text{where }p_{i}\neq0,p_{j}\neq0 \end{matrix}$ |
| 4 | Coarseness | average difference between the center voxel and its neighbourhood and is an indication of the spatial rate of change. A higher value indicates a lower spatial change rate and a locally more uniform texture | $Coarseness=\frac{1}{\sum_{i=1}^{N_{g}} p_{i}s_{i}}$ |
| 5 | Strength | high value menas that an image is with slow change in intensity but more large coarse differences in gray level intensities | $Strength=\frac{\sum_{i=1}^{N_{g}} \sum_{j=1}^{N_{g}} (p_{i}+p_{j})(i-j)^{2}}{\sum_{i=1}^{N_{g}} s_{i}}\text{, where }p_{i}\neq0,p_{j}\neq0$ |

A NGTDM quantifies the difference between a gray value and the average gray value of its neighbours within distance δ.

**Table S1-7 Description of the shape-based features**

| **Index** | **Features name** | **Description** |
| --- | --- | --- |
| 1 | SurfaceArea | The total surface area in the VOI |
| 2 | Volume | the volume of the VOI |
| 3 | SurfaceVolumeRatio | a lower value indicates a more compact (sphere-like) shape |
| 4 | Maximum2DDiameterColumn | the largest pairwise Euclidean distance between surface mesh vertices in the coronal plane |
| 5 | Maximum2DDiameterRow | the largest pairwise Euclidean distance between surface mesh vertices in the sagittal plane |
| 6 | Maximum2DDiameterSlice | the largest pairwise Euclidean distance between surface mesh vertices in the axial plane |
| 7 | Maximum3DDiameter | largest pairwise Euclidean distance between surface mesh vertices |
| 8 | LeastAxis | smallest axis length of the VOI-enclosing ellipsoid |
| 9 | MajorAxis | the largest axis length of the VOI-enclosing ellipsoid |
| 10 | MinorAxis | the second-largest axis length of the VOI-enclosing ellipsoid |
| 11 | Elongation | the root square value of ration of Minor Axis to Major axis, and bigger value means the shape is more circle-like |
| 12 | Flatness | the root square value of ration of Least Axis to Major axis. bigger value means the shape is more non-flat |
| 13 | Sphericity | the roundness of the shape of the VOI relative to a sphere |
