# Supplementary material for: Radiomic Features of the Nigrosome-1 Region of the Substantia Nigra: Using Quantitative Susceptibility Mapping to Assist the Diagnosis of Idiopathic Parkinson's Disease
Source: Front Aging Neurosci. 2019 Jul 16;11:167. doi: 10.3389/fnagi.2019.00167 (PMC6648885; doi:10.3389/fnagi.2019.00167)
Supplement: Supplementary file 2 [file Table_2.DOCX]

**Supplementary S2**

**Table S2 Unpaired t-test results of all the selected features**

| **Index** | **features name** | **IPD** | **HCs** | **p-value** |
| --- | --- | --- | --- | --- |
| **1** | firstorder_10Percentile | 0.023±0.007 | 0.015±0.009 | 1.49E-9 |
| **2** | firstorder_Median | 0.076±0.016 | 0.066±0.015 | 4.10E-5 |
| **3** | glszm_GrayLevelNonUniformity | 5.769±2.442 | 7.583±2.707 | 1.40E-5 |
| **4** | glszm_SmallAreaLowGrayLevelEmphasis | 0.277±0.119 | 0.242±0.109 | 5.70E-2 |
| **5** | glrlm_LongRunLowGrayLevelEmphasis | 0.420±0.133 | 0.546±0.312 | 1.00E-3 |
| **6** | glcm_Correlation | 0.439±0.088 | 0.525±0.067 | 1.14E-10 |
| **7** | gldm_DependenceEntropy | 5.446±0.271 | 5.729±0.198 | 2.06E-12 |
| **8** | gldm_DependenceVariance | 17.871±5.109 | 25.276±6.506 | 1.26E-13 |
| **9** | glrlm_RunEntropy | 3.019±0.212 | 3.212±0.153 | 3.55E-10 |
| **10** | firstorder_Minimum | -0.022±0.011 | -0.039±0.014 | 5.53E-15 |
| **11** | glcm_Imc1 | -0.128±0.026 | -0.169±0.040 | 6.38E-12 |
| **12** | gldm_GrayLevelNonUniformity | 215.113±45.501 | 273.045±52.497 | 3.47E-12 |
| **13** | glrlm_LongRunEmphasis | 3.070±0.533 | 3.815±0.925 | 9.42E-9 |
| **14** | gldm_LargeDependenceEmphasis | 96.868±24.193 | 131.758±35.132 | 2.83E-11 |
| **15** | glrlm_RunLengthNonUniformityNormalized | 0.548±0.055 | 0.484±0.059 | 4.71E-11 |
| **16** | shape_Volume | 519.514±128.743 | 629.073±129.558 | 2.46E-7 |
| **17** | shape_SurfaceArea | 587.813±102.299 | 668.297±109.752 | 3.00E-6 |
| **18** | glrlm_RunVariance | 0.780±0.199 | 1.055±0.323 | 2.84E-9 |
| **19** | shape_Maximum2DDiameterColumn | 26.800±2.524 | 27.928±2.058 | 2.00E-3 |
| **20** | glszm_ZoneEntropy | 3.428±0.452 | 3.621±0.354 | 3.00E-3 |
| **21** | firstorder_Kurtosis | 2.417±0.321 | 2.519±0.286 | 3.50E-2 |
| **22** | shape_LeastAxis | 4.006±0.299 | 4.133±0.169 | 1.00E-3 |
| **23** | gldm_DependenceNonUniformityNormalized | 0.075±0.013 | 0.060±0.008 | 3.68E-15 |
| **24** | gldm_LargeDependenceLowGrayLevelEmphasis | 11.043±4.140 | 16.162±9.622 | 4.00E-5 |
| **25** | glszm_SmallAreaEmphasis | 0.403±0.094 | 0.385±0.106 | 2.35E-1 |
| **26** | shape_Maximum2DDiameterSlice | 27.402±2.533 | 28.483±2.167 | 4.00E-3 |
| **27** | shape_MajorAxis | 33.441±3.414 | 35.665±3.167 | 3.20E-5 |
| **28** | glrlm_GrayLevelNonUniformity | 147.846±25.541 | 168.337±27.700 | 2.00E-6 |
| **29** | glszm_SizeZoneNonUniformity | 3.985±1.939 | 4.481±2.458 | 1.59E-1 |
| **30** | glcm_Idn | 0.903±0.012 | 0.916±0.012 | 9.19E-11 |
| **31** | firstorder_Skewness | 0.226±0.188 | 0.105±0.238 | 4.36E-4 |
| **32** | glcm_Imc2 | 0.558±0.079 | 0.631±0.070 | 5.12E-9 |
| **33** | firstorder_RootMeanSquared | 0.090±0.018 | 0.080±0.016 | 3.44E-4 |
| **34** | shape_MinorAxis | 12.558±1.451 | 13.245±1.485 | 3.00E-3 |
| **35** | glcm_DifferenceVariance | 0.422±0.099 | 0.376±0.083 | 1.00E-3 |
| **36** | shape_Maximum2DDiameterRow | 13.683±1.988 | 14.636±1.952 | 2.00E-3 |
| **37** | glcm_InverseVariance | 0.470±0.031 | 0.426±0.057 | 1.42E-8 |
| **38** | firstorder_Entropy | 1.830±0.277 | 1.809±0.254 | 6.14E-1 |
| **39** | glszm_LargeAreaHighGrayLevelEmphasis | 39374.542±15712.722 | 51838.112±22547.567 | 6.10E-5 |
| **40** | gldm_LowGrayLevelEmphasis | 0.150±0.034 | 0.157±0.054 | 3.72E-1 |
